# Quantitative Evaluation of Aerosols Produced in the Dental Office during Caries Treatment: A Randomized Clinical Trial

**DOI:** 10.3390/jcm12144597

**Published:** 2023-07-10

**Authors:** Jacek Matys, Tomasz Gedrange, Marzena Dominiak, Kinga Grzech-Leśniak

**Affiliations:** 1 Oral Surgery Department, Wroclaw Medical University, Krakowska 26, 50-425 Wroclaw, Poland; tomasz.gedrange@umw.edu.pl (T.G.); marzena.dominiak@umw.edu.pl (M.D.); kinga.grzech-lesniak@umw.edu.pl (K.G.-L.); 2 Department of Orthodontics, Technische Universitat Dresden, 01307 Dresden, Germany; 3 Department of Periodontics, School of Dentistry, Virginia Commonwealth University, Richmond, VA 23284, USA

**Keywords:** bacteria, contamination, dentistry, hazards, high-volume evacuator

## Abstract

Background: Effective removal of aerosols generated during dental treatment is crucial for maintaining biosafety in dental practice. This study aimed to measure the aerosol amount and the number of aerobic bacteria in the air during caries treatment. Methods: The study involved 50 molar teeth (n = 50) in the mandible in 50 patients divided into two groups based on the type of a high-volume evacuator (HVE); G1 (n = 25) conventional HVE (EM19 EVO, Monoart^®^ Euronda, Vicenza, Italy) and G2 (n = 25) a new, wider, customized HVE. The PC200 laser particle counter (Trotec GmbH, Schwerin, Germany) was used to measure aerosol particles in a range of 0.3–10.0 μm near the operator’s mouth. The study used 60 microbiological plates with a microbiological medium (Columbia Agar with 5% Sheep Blood) to check the number of aerobic bacteria in the air. Results: The mean value of aerosol particles in the G1 group (conventional HVE) was 54,145 ± 7915, while in the G2 group (test, wider evacuator) was lower and amounted to 32,632 ± 1803. (*p* < 0.001). The median total bacteria count in the air per cubic meter in control, G1 (HVE), and G2 (NEW-HVE) groups were 50 [36-60]; 772 [643-881]; 120 [92-139], respectively. (*p* < 0.05). Gram-positive cocci were the predominant bacteria in the plates: *Micrococcus* sp. (50%), *Bacillus* species (36.4%), *Staphylococcus epidermidis* (3.8%), *Staphylococcus saprophyticus* (3.8%). Conclusions: the application of the wider high-volume evacuator increases the air purity during caries treatment as well as the biological safety of a dental office.

## 1. Introduction

Dental rotary instruments which use water-air spray cooling generate aerosols containing various types of microorganisms (viruses, bacteria, fungi) [1]. Reducing a minimal number of aerosols produced during dental treatment is essential, especially for establishing the biological safety of the workplace [2]. Our previous in vitro studies showed that the large amounts of potentially dangerous aerosols are produced during dental treatment which contribute to the risk of pathogen transmission [3,4]. The highest aerosol concentration and risk of transmission of bacteria, viruses, and fungi within the dental office premises, is associated with using all rotary dental instruments (high and low-speed tips) and ultrasonic scalers [3,5,6]. During dental treatment, the pathogen transmission routes are aerosols containing saliva and blood as well as the surface of dental instruments and handpieces [7]. Aerosols have high potential to carry and spread viral/bacterial infection; therefore, dentists should recognize and minimize this risk of microbial transformation, especially when patients are in the incubation period, unaware of their infection, or choose to hide their disease [7].

Based on the Alberta Federation of Labour, medical practitioners are at the highest risk of exposure to SARS-CoV-2 [2]. The time of the COVID-19 pandemic has indicated that dentists should guarantee biosafety with aerosol formation in the dental office during dental treatment [2,8]. Therefore, many procedures have been recommended, such as rinsing the mouth with a disinfectant and treatment only for emergency and pain indications. Aerosols can transmit potentially dangerous infections, spreading other pathogens such as bacteria, fungi, and viruses [2]. The primary infection route is the contact of the bacteria or virus with mucous membranes of the nasal or oral cavity, and eyes [9]. The main characteristic of working in a dental office is prolonged contact with the human mouth and exhaled bioaerosol with water spray from the rotary instruments, which raises the speed and dispersion of aerosols [10,11,12]. Two fundamental sources of contamination in the dental office can be distinguished: respiratory path (breathing, coughing, sneezing) and saliva, blood, and rotary instruments such as high and low-speed handpieces, dental sandblasters, ultrasonic scalers, or lasers [13].

Aerosols are small particles that are suspended in air and can remain airborne for an extended period [5]. Aerosols generated during dental procedures are a significant concern due to their potential to transmit infectious agents, including bacteria, viruses, and fungi. Dental aerosols can range in size from less than one micron to over 100 microns in diameter, with most falling in the range of 10 to 30 microns [3,4].

Conservative dentistry is used in caries treatment when a high-speed contra-angle is applied generates high amount of aerosol [14,15,16]. During the caries treatment, produced aerosol can be divided into the following types [12]; respiratory aerosols or bioaerosols (sneezing, coughing); water spray from rotary instruments; a mixture of water spray with the bioaerosol characterized by a high spreading potential. It should be highlighted that the smaller aerosol particles (especially the diameter below 5 μm) are more hazardous because they can be inhaled into terminal bronchioles and alveoli of the human lungs [10]. The particles with a diameter over 50 μm form splatter patterns and cannot be inhaled and transmitted into the lungs [16]. The main concept of decreasing virus/bacteria transmission in dentistry is to reduce the amount of water spray during different dental procedures [3].

There are several reasons for an increase in bacteria, viruses, and fungi within the dental office premises [17,18]. The dental office is an environment in which patients, staff, and dental equipment interact closely [18]. The use of water-based instruments, aerosols generated during dental procedures, the presence of saliva, blood, and other bodily fluids, and the accumulation of dust and debris contribute to the growth and spread of microorganisms. Inadequate cleaning and disinfecting practices, poor ventilation, and overcrowding can also lead to the proliferation of bacteria, viruses, and fungi [18]. Additionally, patients who attend dental appointments with infectious diseases or compromised immune systems may increase the risk of cross-contamination and transmission of infections within the dental office [7]. Microbiological analysis of air is an important component of environmental monitoring and can be a major health hazard, especially in enclosed environments such as healthcare facilities, hospitals and other public spaces [7,18]. It is also important to monitor air quality in order to determine the risk of airborne transmission of infectious diseases. Microorganisms present in the air can range from viruses and bacteria to fungal spores, so it is important to analyze the air for a wide range of microbial organisms [19].

The key factor in decreasing the airborne transmission seems to be the reduction in the total amount of water spray and aerosol in the air in a dental office [3,4]. The standard systems for removing aerosols during dental procedures utilize a suction system, e.g., a salivary ejector and a high-pressure evacuator [1,3]. However, in a previous in vitro study, we utilized a customized high-pressure handpiece with a broader handpiece to reduce aerosol amount during caries treatment in a dental manikin mouth. The results of our previous in-vitro studies for caries removal with a high-speed contra-angle handpiece demonstrated that broader suction systems (customized evacuators) allowed removing between two and eight times more aerosols when compared with high-volume evacuators and a saliva ejector, respectively [3,4]. The present study is a continuation of our previous research to check whether the results obtained in vitro demonstrate a benefit for the patients treated in vivo in a dental office.

This paper aimed to test a null hypothesis that there is no difference in the number of aerosols and bacteria during caries treatment in a human model.

## 2. Materials and Methods

The study was designed as a randomized and controlled test. The approval of the Local Ethics Committee of Wroclaw Medical University Faculty of Dentistry was obtained (permission number: KB–737/2021), and informed consent following the Helsinki Declaration was obtained from all participating subjects.

### 2.1. Subjects

**Figure 1:**
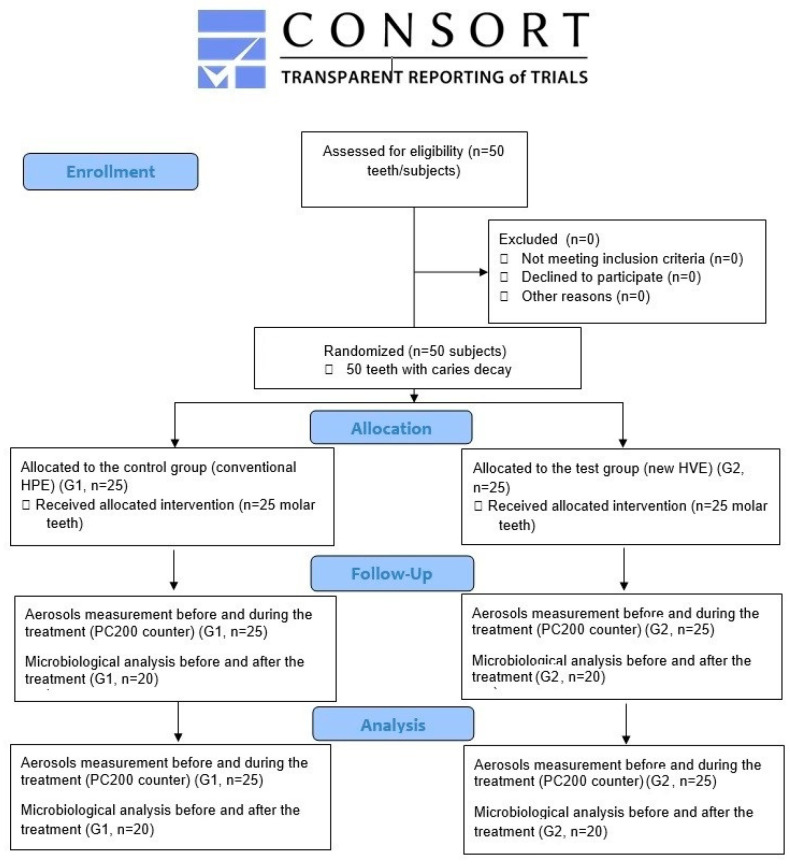
Flowchart of treated subjects based on CONSORT 2010.

The study involved 50 molar teeth in the mandible in 50 patients (28 women and 22 men; age: 34.6 ± 9.3 years) treated due to moderate stage class caries decay (the International Caries Detection and Assessment System; ICDAS 3 and 4). The subjects were chosen for the study under the subsequent inclusion criteria: occurrence of moderate stage class caries decay (ICDAS 3 and 4); all the subjects were not using anti-inflammatory medicines; were non-smokers; had no systemic illnesses; had not used antibiotics in the previous two months; no record of uncompensated diabetes or uncontrolled periodontal disease was observed; each subject had undergone hygienist treatment prior to the study (Figure 1).

### 2.2. Sample Size Calculation

The sample size was calculated to be 25 in each group using G*Power ver. 3.1 (Kiel University, Kiel, Germany) software assuming 80% power of a study, 95% confidence intervals, a level of significance of 0.05, and d = 0.72 based on our previously published studies [3,4].

### 2.3. Caries Treatment Procedure and Study Groups

The study involved 50 molar teeth (n = 50) in the mandible in 50 patients treated due to caries decay. The patients (teeth) were divided into two groups using a random number generator website (www.randomizer.org, accessed on 5 July 2023). In the first group, G1 (n = 25) a conventional high-volume evacuator (HVE) EM19 EVO (Monoart^®^ Euronda, Vicenza, Italy) was used to remove aerosols during caries treatment. In the second G2 (test, n = 25) group, a new, wider, customized high-volume evacuator for aerosol removal was used. Moderate stage class (the International Caries Detection and Assessment System; ICDAS 3 and 4) treatment was done using a round diamond bur (#014) with a high-speed handpiece W&H Synea TA-98LC (W&H, Bürmoos, Austria) at working parameters: 200,000 RPM (revolutions per minute), water cooling: 30 mL/min. The teeth after procedures were restored with the composite material Charisma (Kulzer, Hanau, Germany) according to the manufacturer’s instructions. The time between patients was 120 min.

### 2.4. Aerosol Measurement Protocol

**Figure 2:**
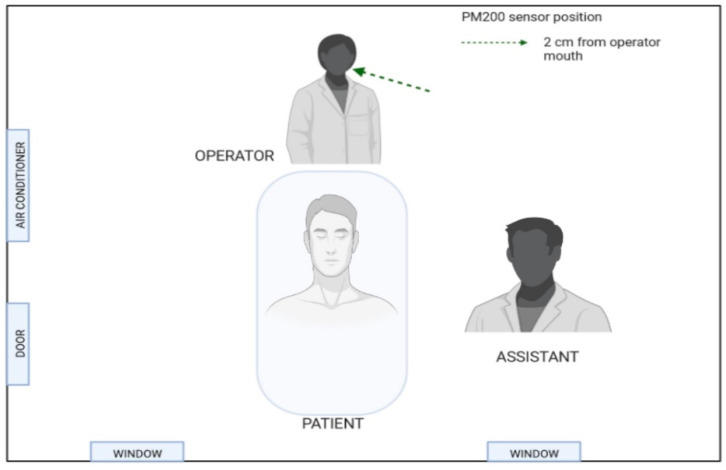
Aerosol particle sensor positions.

The PC200 laser counter (Trotec GmbH, Schwerin, Germany) was utilized to estimate the number of aerosol particles at conducted sites. The nozzle of the counter was placed 2 cm from the operator’s mouth. The PM200 detector (Trotec GmbH, Schwerin, Germany) let us measure six aerosol fractions with a diameter of 0.3–10.0 µm. The PM 200 sensor (Trotec GmbH, Schwerin, Germany) was turned on immediately before each treatment and off at the end of the caries treatment with a high-speed handpiece (drilling procedure). The number of counted particles (various fractions) was summarized, and the mean outcomes were compared among study groups. Our previously published in-vitro studies also described the protocol of aerosol measurements during caries treatment [3,4] (Figure 2).

### 2.5. Testing the Number of Aerobic Bacteria in the Air by the Sedimentation Method

**Figure 3:**
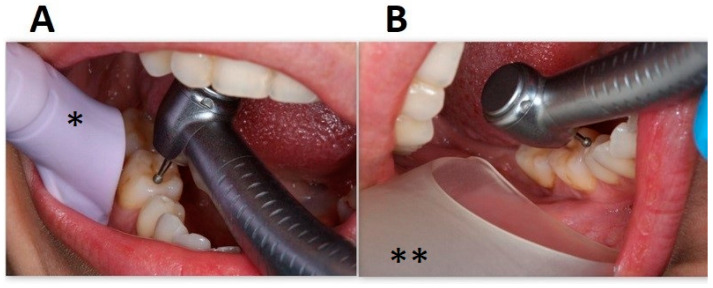
Suction systems used in the study. (**A**). High-volume evacuator EM19 EVO (Monoart^®^ Euronda, Vicenza, Italy). (**B**). Customized high-volume evacuator. * conventional HVE, ** new wider HVE.

The total number of aerobic bacteria in the air of the dental office was carried out by the Koch sedimentation method. The study used 60 Petri dishes with a microbiological medium (Columbia Agar with 5% sheep blood) to check the number of aerobic bacteria. (Figure 3).

Twenty plates were opened 60 min before the study (control group, n = 20) and closed before the start of caries treatment. Next, forty plates were opened at the start of caries treatment with additional use of a conventional evacuator (n = 20) or the new customized evacuator (n = 20) and closed 60 min after the treatment. The measures were performed in the middle of the office, 2 m from the patients’ mouth, at a height of 1 m from the ground [20]. Bacteria were incubated for 48 h at 37 °C. The degree of microbiological contamination based on the total number of CFUs (colony-forming units) in one cubic meter of air was calculated using the formula: L = a × 1000/*π*r^2^ × k.

L—concentration of microbiological impurities in [cfu/m^3^],
a—number of colonies grown on the plate,
r—Petri dish radius [cm],
k—plate exposure time factor, k = t × 1/5, where t—exposure time (min)

In the first stage, bacterial identification was made in compliance with standard protocols, including observing the morphology and using microscopy. The cultured bacteria were analyzed microscopically, taking into account the Gram stain and evaluating the size, form and presence of spores. In the second stage, the bacteria were metabolically characterized using the APIWeb-supported Analytical Profile Index (API) by Biomerieux Inc. (Marcy-l’Étoile, France), and quality control was carried out in accordance with the PN-EN12322 standard.

### 2.6. Spray/Aerosol Evacuators

Two different intraoral suction systems for aerosol removal were used during dental treatment: (A). High-volume evacuator (HVE) EM19 EVO (Monoart^®^ Euronda, Vicenza, Italy). (B). A customized, digitally designed high-volume evacuator. Evacuators were placed at the level of the molar teeth about two centimeters from its buccal side (Figure 3).

### 2.7. The New High-Volume Evacuator Design

**Figure 4:**
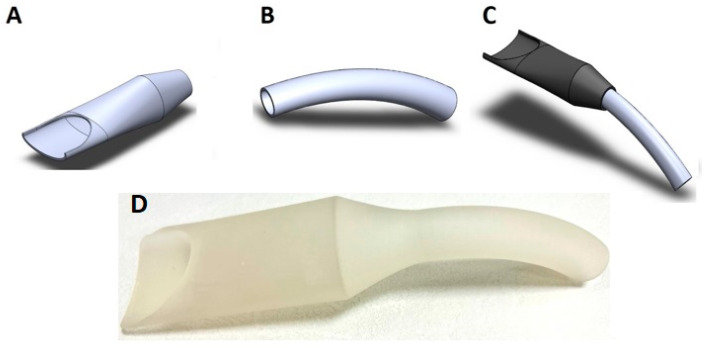
The computer-aided design evacuator. (**A**). Working part. (**B**). Exhaust part. (**C**). The customized high-volume evacuator. (**D**). The printed version of HVE. (Design pattern registered with the European Union Intellectual Property Office under number 008056360-0001).

The new HVE used in the study was designed in AutoCAD software (Autodesk, San Francisco, CA, USA) based on the previous design of the HVE described in the authors’ earlier study [4]. The created 3D images had been exported to STL (Stereo Lithography) file format and were printed using a high-temperature resistance light-curing resin (Siraya Tech, San Gabriel, CA, USA) which has the following mechanical parameters: a heat deflection point (160 °C), liquid density (1.13 g/cm^3^), hardness (Shore D 82), tensile strength at break (63 MPa), elongation at break (6%), Young’s modulus (1000 Mpa). The Creality CR-5060 Pro (Shenzhen Creality 3D Technology Co., Ltd., Shenzhen, China) 3D printing machine was employed. The evacuator was made of 2 permanently connected parts. The wider working part had a width of 30 mm in a vertical projection and a length of 50 mm. The second part of the evacuator, in the shape of a flattened tube, was 10 mm wide and 100 mm long. The total length of the HVE was 150 mm. The wider working part allowed placing a turbine head within it to stay as close as possible to the aerosol-generating nozzle of the dental turbine. The evacuator design was registered with the European Union Intellectual Property Office under number 008056360-0001 (Figure 4).

### 2.8. The Office Air Standardization

All tests were conducted in a dental office size of 20 square meters. During the study, windows and doors were locked, and the air conditioning was switched off. The steady value of aerosols in the room was maintained in a range of 28.000–30.000 using the air cleaner (NV1050, Novaerus, Dublin, Ireland) with 800 m^3^ per hour air exchange before each procedure. Control measures were done every minute while the purifier was on. Each treatment procedure was performed after the office air standardization within the assumed range. The control measurement of particles in the room was evaluated after positioning the sensor in the center of the office. The average time to clean the air to the required level was approximately five minutes.

### 2.9. Statistical Analysis

The Kolmogorov-Smirnov test was used to check the data normality. The results of aerosol amount measured by particle counter PM 200 during caries treatments were assessed using a *t*-test for independent groups. The total bacteria count (cfu/m^3^) results were compared with the ANOVA Kruskala-Wallisa analysis. The occurrence of homogeneity of variance was assessed using Levene’s test. Statistica software, ver.13.3.721.1 (StatSoft, Tulsa, OK, USA) was used for statistical analysis. Values below *p* = 0.05 were considered to be statistically significant.

## 3. Results

### 3.1. The Number of Aerosol Particles during Caries Treatment

**Figure 5:**
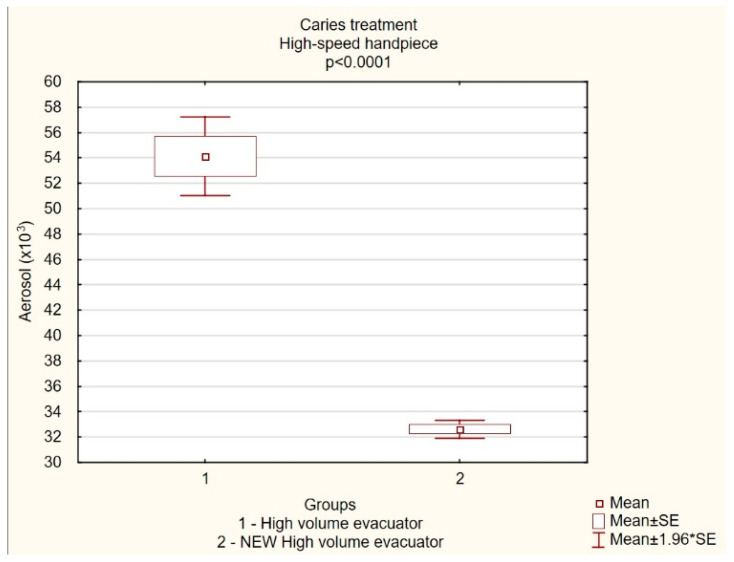
The level of aerosol particles (×10^3^) measured operator mouth (mean ± SD) during caries treatment with conventional high-volume evacuator or a new customized high-volume evacuator.

The aerosol level measured at the operator’s mouth was significantly lower for the wider customized high-volume evacuator compared to the conventional, standard-sized evacuator. (*p* < 0.001) The mean value of aerosol particles in the G1 group (conventional HVE) was 54,145 ± 7915, while in the G2 group (test, wider evacuator) it was lower and amounted to 32,632 ± 1803. The results comparison of the two groups indicated a 40% reduction of aerosol levels during caries treatment when a wider evacuator was applied with a high-speed turbine (Figure 5).

### 3.2. The Number of Aerobic Bacteria in the Air of a Dental Office during Caries Treatment

The median total bacteria count in the air per cubic meter in control, G1 (HVE), and G2 (NEW-HVE) groups were 50 [36-60]; 772 [643-881]; 120 [92-139], respectively. The number of bacteria measured in the air of the dental office per cubic meters showed a significant increase in bacteria CFU level during caries treatment using a dental turbine with a conventional evacuator (G1, *p* < 0.0000) and the new wider suction system (G2, *p* < 0.0011). However, the results of the study showed significantly less TBC calculated in CFU/m^3^ for the wider evacuator in contrast to the conventional one. (*p* < 0.0008) Moreover, the comparison of the results between both suction systems indicated an 84.5% reduction of aerosol levels during caries treatment when a wider evacuator was applied with a high-speed turbine (Table 1).

The qualitative analysis of the microbiological assessment of the air indicated the greatest variety of bacterial strains for the plates distributed during the use of a conventional high-volume evacuator. However, some samples in the control group also showed the presence of specific bacterial strains. Gram-positive cocci were the predominant bacteria observed in all the plates: *Micrococcus* sp. (50%), *Bacillus species* (36.4%), *Staphylococcus epidermidis* (3.8%), *Staphylococcus saprophyticus* (3.8%) (Table 2).

## 4. Discussion

Air purity in a dental office is crucial in decreasing the risk of microbial transmission [21]. The last two years of the COVID-19 pandemic have shown that special efforts should be made to maintain biological safety for patients and medical staff. Using systems for air decontamination, protective masks, or surface disinfection increases the safety and air purity in the treatment room [3,4,14]. However, the essential element in reducing the risk of bioaerosol transmission from the respiratory tract during dental treatment is removing the mixture of bioaerosol and cooling spray directly within the oral cavity [22]. The study aimed to remove the aerosol generated during dental treatment in the oral cavity before transferring it to the dental office environment. This procedure allowed significant reduction in the microbiological hazards present in the air of the dental office. Our present study showed a decrease in the mean aerosol amount when using a wider evacuator compared to conventional suction system. Moreover, microbiological evaluation of a dental office air purity showed a significant reduction of aerosol levels during caries treatment when a wider evacuator was applied with a high-speed turbine. The results of the present randomized controlled trial confirmed other previously published in vitro studies [3,4,21].

The primary aim of the study was to test a null hypothesis that there is no difference in aerosol amount produced during caries treatment with a bur on a dental turbine when using a conventional or wider customized high-pressure evacuator. The findings of the in vivo study proved that applying a wider customized high-volume evacuator during caries treatment significantly decreased the aerosol level measured at the operator’s mouth compared with conventional HVE. These findings were similar to our previously published in vitro study [3,4]. In recent scientific literature, other authors proved the efficiency of applying a high-volume evacuator to decrease the level of aerosols in the dental office [11,16,22,23]; however, our present study is the first in vivo trial comparing the efficiency of different size evacuators. In studies by Harrel et al. [16] and Jacks [11], the authors obtained significantly better efficiency of HVE as compared with a salivary ejector for aerosol removal in a dental office. These results are consistent with our previous in vitro studies. However, the current study on the use of the wider HVE indicates that increasing the diameter of the evacuator using the same suction pressure reduces aerosol levels during caries treatment an additional 40% in contrast with conventional HVE. Also, in vivo results of a study by Nulty et al. [22] were consistent with our present outcomes and also proved the aerosol reduction generated during dental procedures. However, Nulty et al. [22] utilized external suction systems to remove aerosol particles.

There are a number of serious public health concerns worldwide due to the spread of bacterial infections [24,25,26]. The study’s second aim was to assess the number of aerobic bacteria in the air of a dental office during caries treatment. We found that the number of bacteria measured in the CFU level in the dental office air per cubic meter increased for both HVEs compared to the initial control level. However, the results of the total bacterial count for the wider evacuator showed a 84.5% reduction of aerosol levels during caries treatment compared to the conventional suction handpiece measured two meters from the patients’ mouths. In a study by Manarte-Monteiro et al. [20], the authors demonstrated an increase in the airborne bacterial load at a distance of 0.5 m and 2 m during endodontic treatment. Furthermore, Rautemaa et al. [27] reported significant room contamination at 0–2 m distance sampled when high-speed instruments were used (mean 970 colony-forming units/m^2^/h). The maximal median results of bacterial count in our present study were obtained for conventional HVE and amounted to 772 [643-881] CFU/m^3^ after one hour. In turn, Szymańska [28] showed that the airborne concentration of fungi ranged from 4 × 101 CFU/m^3^ to 34 × 101 CFU/m^3^ during conservative dental treatment.

Additionally, gram-positive cocci were the predominant bacteria in the plates; *Micrococcus* sp. (50%), *Bacillus* species (36.4%), *Staphylococcus epidermidis* (3.8%), *Staphylococcus saprophyticus* (3.8%). Among isolated bacteria were Micrococcus species which are frequently present on human skin, and is the main reservoir for these strains [29]. Also, *Staphylococcus* species are a typical associate of the body’s microbiota, often found on the skin and in the upper respiratory tract [30]. In turn, Bacillus species were second in order of frequency in the samples in our study. Most of these *Bacillus* strains have no pathogenic potential and have never been associated with human infections [31]. Similar findings to ours were obtained in a study by Mirhoseini et al. [32], who also found a prevalent number of gram-positive Cocci in all isolates. These results were confirmed by Adhikari et al. [33], Kimmerle et al. [34] and Manarte-Monteiro et al. [20]. In their studies [20,33,34], the most isolated bacteria were *Micrococcus* sp., *Bacillus* sp., *Streptococcus* sp., and *Staphylococcus* sp., which were also proven in our present study.

Several studies have investigated bacterial content in indoor air of dental and medical offices. In a study conducted in Iran by Mirhoseini et al. [32] the authors collected indoor air samples from three dental clinics and identified the bacteria using culture-based methods. The results showed that the most dominant bacterial species were *Micrococcus*, *Bacillus*, *Streptococcus*, *Staphylococcus*, *Penicillium*, *Cladosporium*, *Aspergillus*, *Rhizopus*, and *Alternaria* [32]. Similarly, another study conducted in Portugal by Manarte-Monteiro et al. [20] identified *Micrococcus* sp., *Staphylococcus* sp. and *Streptococcus* sp. from indoor air samples of a dental clinic. In a study conducted in the USA by Adhikari et al. [33], the authors isolated bacteria during different stages of the dental cleaning procedures from the indoor air of several dental clinic rooms and identified the most abundant species such as *Psychrobacter* sp. (including *P. pulmonis*, and *P. faecalis*), *Streptococcus* sp. (including *S. thermophiles*, *S. parasanguinis*, and *S. oralis*), *Pseudomonas* sp. (including *P. graminis*). These studies indicate that the bacterial content of indoor air in dental clinics can vary depending on the location and time of sampling [20,32,33]. Our present study conducted in Poland showed that the most dominant bacterial species were *Micrococcus* sp. (50%) and *Bacillus* species (36.4%). However, in our study, contrary to the indicated authors, the presence of *Staphylococcus epidermidis* and *Staphylococcus saprophyticus* was observed in 3.8% of samples. However, the results may be related to differences in the composition of the bacterial microflora of patients in different geographic regions (environments).

Last but not least, saliva ejectors and standard high-volume evacuators are used for fluids removal from the oral cavity during various dental treatments [3,4,10,19,35]. The saliva ejector removes fluids from the oral cavity with negligible efficiency in removing aerosols [3,4]. Numerous studies indicated that the standard high-volume evacuator in contrast to salivary ejector removes fluids and aerosol from the oral cavity more efficiently [3,4,11,16,22]. However, its small width does not allow the aerosol to be removed with high efficiency when using a dental turbine during the treatment of caries or preparing teeth for prosthetic reconstruction [4]. The use of a larger diameter evacuator with an indentation allows getting closer to the head of the turbine during its operation and removing aerosol within the oral cavity, which helps to keep the number of aerosols in the dental office at a constant initial level, as was indicated by our current and previous in-vitro studies [3,4]. 3D technology, which allows for precise design and subsequent production of dental instruments, was used in our research [36]. The wider evacuator used in the study was printed with a high-temperature resistance resin with heat deflection point of 160 °C. The resin was made by combining thermosetting resins with high-temperature resistant fillers and additives such as ceramic fibers, graphite, carbon fibers, nanoparticles, silica, and metal powders [37]. The HVE, which was printed using high-temperature resin, allowed it to be sterilized in an autoclave set to a maximum temperature of 132 °C and reused several times.

The present study has several limitations. First, the study’s sample size was limited to 50 subjects. Secondly, in the study, we assessed only the teeth with moderate caries (ICDAS 3 and 4). The caries treatment of teeth with deep caries lesion (ICDAS 5 and 6) located in areas of the oral cavity that are difficult to access for treatment should be included due to the risk of extending the procedure time and, consequently, a possible increase in the level of aerosols in the dental office. Thirdly, the microbiological examination did not assess the presence of viruses and fungi in the dental office. Further studies should be conducted to investigate aerosols produced during direct [38] or indirect [39] restorative procedures in anterior teeth where HVE have different positions in respect to posterior teeth and less soft tissue protection. Also, additional measurements should be conducted to investigate the influence of other devices for caries treatment, such as lasers and dental sandblasters, or to assess the effect of using different evacuator shapes on the aerosol amount in a dental office.

## 5. Conclusions

Dental caries treatment using a classic high-speed handpiece generates a large number of aerosols, which can remain in the air of a dental office for several hours and pose the possibility of transmitting infections between patients and medical personnel. The carried-out study demonstrated a high reduction of aerosols when using the wider high-volume evacuators compared to a standard shape HVE. Moreover, compared to conventional, a significant reduction in the total bacterial count calculated in CFU/m^3^ was found for wider evacuators. The wider high-volume evacuator application during caries treatment increased the air purity and the biological safety of the dental office.

## Figures and Tables

**Table 1:** The total bacteria count (cfu/m^3^) measured in the center of the dental office (median).

Groups	ANOVA Kruskala-Wallisa; H (2, N = 60) = 52, 08879 *p* = 0.0000			
	n	Median	Lower-Top Quartiles []	*p* Value
Control (C)	20	50	[36-60]	C vs. G1 = 0.0000
HVE (G1)	20	772	[643-881]	C vs. G3 = 0.0011
NEW-HVE (G2)	20	120	[92-139]	G1 vs. G2 = 0.0008

HVE—high volume evacuator, NEW-HVE—a new high volume evacuator, TBC—total bacteria count, cfu—colony-forming unit, SD—standard deviation.

**Table 2:** Bacterial strains recognized with microbiological analysis. The occurrence of individual strains of bacteria in the samples per total number of samples was described in square brackets.

	Control (C, n = 20)	HVE (G1, n = 20)	NEW-HVE (G2, n = 20)
Bacterial Species	*Micrococcus* species [14/20]	*Micrococcus* species (19/20) *Bacillus* species (19/20) *Staphylococcus epidermidis* (6/20) *Staphylococcus saprophyticus* (4/20) *Staphylococcus arlettae* (4/20)	*Micrococcus* species (19/20) *Bacillus* species (18/20)

## Data Availability

The raw data are available upon request from the corresponding author.
